# The application of photodynamic therapy in plastic and reconstructive surgery

**DOI:** 10.3389/fchem.2022.967312

**Published:** 2022-07-22

**Authors:** Min Wu, Xiaoyu Huang, Lu Gao, Guoyu Zhou, Feng Xie

**Affiliations:** ^1^ Department of Plastic and Reconstructive Surgery, School of Medicine, Shanghai Ninth People’s Hospital, Shanghai Jiao Tong University, Shanghai, China; ^2^ School of Biomedical Engineering, Shanghai Jiao Tong University, Shanghai, China; ^3^ Department of Oral and Maxillofacial-Head Neck Oncology, School of Medicine, Shanghai Ninth People’s Hospital, Shanghai Jiao Tong University, Shanghai, China

**Keywords:** photodynamic therapy, plastic and reconstructive surgery, clinical application, tumor, photosensitizer

## Abstract

Photodynamic therapy (PDT) is a modern clinical treatment paradigm with the advantages of high selectivity, non-invasiveness, rare side-effect, no obvious drug resistance and easy combination with other therapies. These features have endowed PDT with high focus and application prospects. Studies of photodynamic therapy have been expanded in a lot of biomedical and clinical fields, especially Plastic and Reconstructive Surgery (PRS) the author major in. In this review, we emphasize the mechanism and advances in PDT related to the PRS applications including benign pigmented lesions, vascular malformations, inflammatory lesions, tumor and others. Besides, combined with clinical data analysis, the limitation of PDT and current issues that need to be addressed in the field of PRS have also been discussed. At last, a comprehensive discussion and outlooking represent future progress of PDT in PRS.

## 1 Introduction

Compared with traditional surgery, as a result of non-invasive and safe treatment modality, laser-treatment has been widely used in the field of plastic and reconstructive surgery (PRS) to cure lots of diseases, including vascular lesions (e.g., vascular malformation, haemangioma), pigmentary diseases (naevi of Ota), benign proliferation, premalignant conditions and so on. Photodynamic therapy (PDT) is a clinically approved therapeutic procedure that can exert laser irradiation to generate cytotoxic activity against malignant cells. Besides diseases mentioned before, many cosmetic problems such as acne vulgaris and photorejuvenation can also be treated with photodynamic therapy. In this review, we introduce mechanism, principle and all representative researches of PDT for the mentioned applications in the field of plastic and reconstructive surgery. The limitations and development prospects of PDT for clinical application are also discussed.

## 2 Mechanism and principle of photodynamic therapy

Photodynamic therapy as a modern and non-invasive treatment method has possessed desirable therapeutic outcome and the possibility of the parallel application of PDT with other therapeutic protocols, which make it more commonly used in many fields of medicine (Luo et al., 2017). During the process, PDT based on photosensitive compound-photosensitizer (PS) combines the photophysical and photochemical processes to bring about biological effect in pathological tissues. In response to specific wavelengths of light, PS can generate reactive oxygen species (ROS). The origins of PDT can be traced back to ancient Egypt, where photosensitizing plant pigment extracts were applied to the skin and exposed to sunlight as a treatment for psoriasis (Daniell and Hill, 1991). In recent decades, PDT has developed rapidly and achieved progressive results.

### 2.1 Mechanism

**FIGURE 1:**
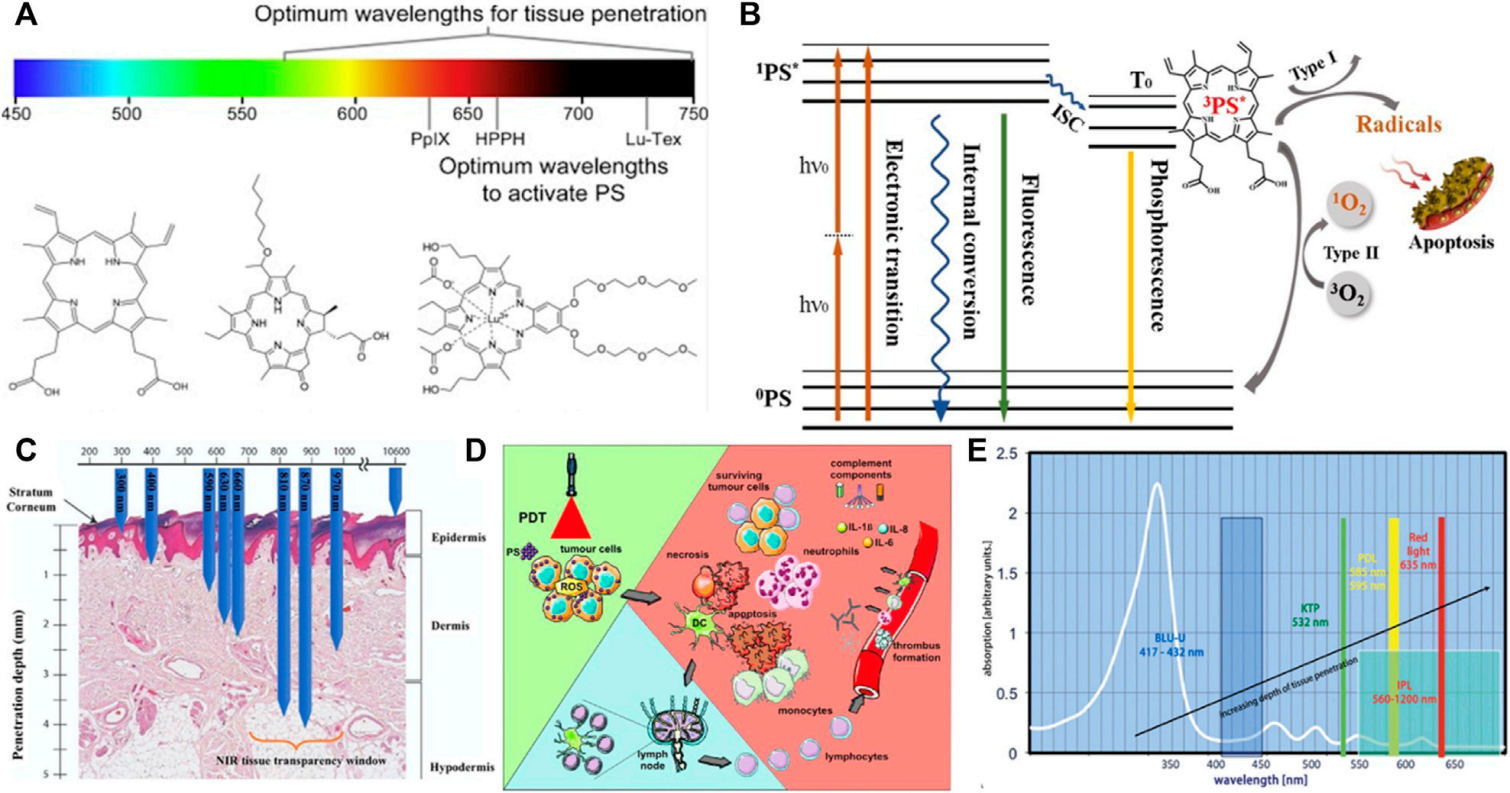
Fundamentals of Photodynamic therapy. **(A)** Visible and near infra-red light spectrum showing the wavelengths (nm) of maximum tissue penetration by light (above) and absorbance maxima of selected photosensitizers (below); Chemical structures of common photosensitizers including PpIX, HPPH, Lu-Tex from Dobson et al. (2018). **(B)** Jablonski level diagram and two approaches of photodynamic therapy. **(C)** Penetration depth of different wavelengths of laser in tissue, UV, ultraviolet light; VIS, visible light; NIR, near infrared light. **(D)** PDT-induced effects. **(E)** PpIX absorption *in vivo* for different kinds of light source from Ozog et al. (2016).

The mechanism of photodynamic therapy involves three important factors, photosensitizer (PS); light with the appropriate wavelength; oxygen dissolved in the cells (Allison and Moghissi, 2013a), which produce the desired effects within pathological tissues only by mutual interactions. As shown in Figure 1B, there are two main mechanisms of the photodynamic reaction, both of which rely on oxygen molecules inside cells. The first stage of both mechanisms is similar. Exposure to laser irradiation, photosensitizer can absorb photon and then be excited from the singlet ground state S° into the excited singlet state S^1^. Though intersystem crossing (ISC) process, the excited triplet state T^1^ of PS is formed (Castano et al., 2005; Robertson et al., 2009).

The second stage can be divided two routes, where the T^1^ state of PS either directly reacts with cellular substrates by electron transfer to produce ROS (Type I reaction) or transfers energy to molecular oxygen, leading to the generation of highly reactive singlet oxygen (^1^O_2_) (Type II reaction) (Maiya, 2000). The singlet oxygen is considered to be the most damaging species as it reacts with biological molecules like unsaturated lipids, a amino acids of proteins (tryptophan, histidine, methionine) that are main components of cell and nuclear membranes (Foote, 1991). Thus, photosensitizers can kill tumor cells using type I reaction (corroles) or type II (Photofrin II) reaction or by simultaneously using both types of reaction mechanisms (fullerenes, hypericin) (Figure 1D). 

### 2.2 Photosensitizers

Photosensitizers, primarily derived from natural or synthetic chemicals, can transfer photon energy to neighboring molecules, importantly to dissolved oxygen. According to clinical analysis results, an ideal PS is required to possess four important characteristics, including nontoxicity before photoactivation, hydrophilicity, suitable excitation wavelength activated by a clinically common light. What’s more, PS can reliably generate a photodynamic reaction (PDR) (Allison and Moghissi, 2013b). Haematoporphyrin derivative (HpD) and Photofrin as the first-generation representative photosensitizers have been clinically approved and developed rapidly. The commercially available product, porfimer sodium marketed under the tradename Photofrin, was approved for the early stage of human lung cancer treatment in 1998 and for Barrett’s oesophagus in 2003. Clinically, two main problems occurred. The absorption peak wavelength of Photofrin is too short to allow deep penetration of light in tissue. Another problem is that cutaneous photosensitivity lasting up to 6 weeks (Zhu and Finlay, 2008). These limitations accelerate the development of a second generation of photosensitizers with improved efficiency of ROS generation, more rapid clearance, fewer side effects and absorption peaks at longer wavelengths (>630 nm), where the tissue penetration of light is deeper. For instance, 5-aminolaevulinic acid (ALA) is a natural pro-photosensitizer and precursor for the biosynthesis of the haem molecule. In the presence of excess ALA, tumor cells that combine high ALA uptake with low PpIX destruction will accumulate PpIX photosensitizer (Collaud et al., 2004). In the field of plastic and reconstructive surgery, ALA has been used for the treatment of basal cell carcinoma (Kim et al., 2012), squamous cell carcinoma (SCC) and other head and neck tumors (Hage et al., 2007). There are also many other second-generation photosensitizers, such as m-tetrahydroxophenyl chlorine, 2-(1-hexyloxyethyl)-2-devinyl pyropheophorbide-a, palladium bacteriopheophorbide and its more water-soluble monolysotaurine derivative (Figure 1A).

Following list three generations of photosensitizers and their characteristics (Table 1).

**TABLE 1:** A summary of kinds of photosensitizers and their characteristics.

Types	Classification	Typical samples	Characteristics	References
1st generation photosensitizers	Porphyrin derivatives	*HpD (hematoporphyrin derivative)*	Disadvantages	Zhu and Finlay (2008), Allison and Moghissi (2013b)
			Absorption peak wavelength too short to allow deep penetration of light in tissue	
		*Photofrin*	Cutaneous photosensitivity lasts too long	
2nd generation photosensitizers	Hematoporphyrin derivatives	*ALA (5-aminolevulinic acid)*	Advantages	Collaud et al. (2004), Kim et al. (2012)
			Higher chemical purity	
		*BPD (benzoporphyrin derivative)*	Higher yield of singlet oxygen formation	
	Chlorine derivatives	*Texaphyrins*	Better penetration to deeply located tissues	
		*Thiopurine derivatives*	Maximum absorption in the wavelength range 650–800 nm	
	Dyes family	*Bacteriochlorin analogues*	Fewer side effects	
		*Phthalocyanine*	Disadvantages	
			Poor solubility in water	
			Limited in intravenous administration	
3rd generation photosensitizers	NIR-absorbance PS	*polymers*	Development	Hage et al. (2007)
			Higher affinity to the tumor tissue, less damage to the surrounding healthy tissues	
		*Liposomes*		

### 2.3 Light source

Light sources including coherent and incoherent light play an important role in PDT process. PpIX has a strong absorption peak between 405 and 415 nm located in soret band and several smaller Q bands from 500 to 630 nm with the last peak at 635 nm. Blue light is commonly used to efficiently excites PpIX and the most widely available fluorescent lamp light source is the Blu-U (DUSA) with a peak emittance at 417 ± 5 nm (Goldman et al., 2013). Because of its relatively short wavelength, blue light penetrates about 2 mm, whereas red light (635 nm) is used for thicker lesions because it has a greater than 2 mm penetration (Moan et al., 1996). The 635 nm wavelength targets the last Q band, because red light does not excite PpIX as efficiently as blue light, a higher fluence (dose) is needed (Van der veen et al., 1996). However, the consensus group noted that clinically blue light is effective in some instances where you would only expect red light to work. This may be related to incomplete knowledge as to the number of photons needed to activate aminolevulinic acid. The reported penetration depths for various wavelengths of light reflect the point at which 50% of the photons have penetrated and the depth of an activity of the remaining photons is unclear with either red or blue light. For instance, a 488 nm argon ion laser has 200 μm of tissue penetration compared with a 694 nm ruby laser which has a 1200 μm penetration depth of 50% of the photons in white skin (Arndt et al., 1997). Intense pulsed light (IPL) is a source of incoherent light, which emits a radiation spectrum from approximately 500–1,200 nm (Star et al., 2002). This light source is particularly useful in photorejuvenation and collagen. Light-emitting diodes (LEDs) provide a narrower spectrum of light irradiation, usually in a 20–50 nm bandwidth. Daylight PDT is being increasingly researched and used, particularly in Europe (Smith et al., 2003). Combined with minimal incubation time, daylight PDT produces results with little to no discomfort to patients, no equipment is needed and patient time in clinicians’ office is minimized. There’s also some challenges include determining and standardizing exposure times for various latitudes and seasonal light variances (Figures 1C,E).

PDT has been successfully used in dermatology, oncology, gynecology and urology. The focus of this review is application of PDT in plastic and reconstructive surgery.

### 2.4 Novel technologies of PDT

There are more and more new technologies in the field of PDT. In 2017, Professor Junle Qu’s team of Shenzhen University published a paper, proposing two-photon photodynamic therapy (TP-PDT) (Gu et al., 2017). The principle of TP-PDT is that the photosensitizer simultaneously absorbs two long wavelength photon transitions to the single excited state, resulting in electron transfer or energy transfer and the generation of reactive oxygen species. Compared with single-photon PDT, TP-PDT has the following two outstanding advantages (Shen et al., 2016; Sun et al., 2017): firstly, the excitation wavelength of TP-PDT is located in the near-infrared band of biological transmission window (800–1100 nm), thus solving the poor weaving penetration problem of single-photon so that it is suitable for deep treatment. Secondly, two-photon therapy can be precisely controlled within the range of λ3 (λ is the wavelength of excitation light) (Shen et al., 2016), with higher spatial selectivity and less damage to normal tissues. It is found that PSs with long wavelength absorption (700–1700 nm) is difficult because PSs requires a high energy triplet state to ensure effective energy transfer from the triplet PSs to oxygen molecules. However, another method to shift the excitation wavelength of PSs to the long-wave region is two-photon excitation. Compared with single-photon excitation, two-photon excitation photodynamic therapy can not only increase the therapeutic depth of light, but also better control the activation of PSs in the process of 3-Dimentional space PDT, thus improving the therapeutic accuracy. This key characteristic of two-photon photodynamic is attributed to the excitation power dependence of two-photon fluorescence, that is, only the focused high-intensity pulsed laser can induce the two-photon absorption process at the focal point. This feature makes two-photon photodynamic therapy having great potential for precision treatment. Professor M. G. Bobo used porous silica coated with a photosensitization agent with a two-photon absorption cross section of 1200 GM and a 760 nm fs laser to treat tumors at 4 mm subcutaneously, reducing tumor weight by 70% and inhibiting tumor metastasis (Gary-Bobo et al., 2011). Professor Ying Gu’s research group used Bis(Arylidene)cycloalkanone, a photosensitizer to perform two-photon photodynamic therapy, effectively sealing blood vessels and treating tumors (Zou et al., 2015). In 2017, Pengfei Wang from Chinese Academy of Sciences introduced multimodal polymer nanoparticles (PNPs) as photosensitizers for two-photon fluorescence imaging and two-photon excitation (Guo et al., 2017). Professor Ricardas in University of Munich once conducted a cell-level exploration of two-photon photodynamic therapy for human malignant glioma C6 cells and added 5-ALA for culture after near-infrared irradiation, proving the feasibility of two-photon excitation 5-ALA therapy.

Besides TP-PDT, there are many other techniques such as metronomic PDT (mPDT) (Lilge et al., 2000), PDT molecular beacons (MBs) (Chen et al., 2004), nanoparticles (NP) (Wilson et al., 2006) and photochemical internalization (Berg et al., 1999).

## 3 Applications in the field of plastic and constructive surgery

As we know, plastic and reconstructive surgery intervenes from head to toe, in all age groups and both sexes. Its role in conditions such as the aging face or reimplantation is undoubtedly well understood by the public. PRS has equally important applications such as tumor (benign or malignant) and trauma. There are many branches in the field of PRS, including pigmented lesions, tumor, trauma, rejuvenation, rhinoplasty, keloid and so on. We can solve these problems with surgery, which is the most traditional technology and also use other technologies. PDT is one of the most essential treatments for many kinds of lesions in the field of PRS. Following we list them in various categories.

### 3.1 Benign pigmented lesions

Q-Switched dye lasers have become useful for treating pigmented skin lesions (Anderson, 1996). People other than Celtics have a minimum to moderate amount of pigment in the basal layer of the *epidermis*, which is enough to cause epidermal damage upon absorbing laser energy. Photodynamic therapy (PDT) permits two ways to achieve selectivity by spatial localization of the illumination and by preferential targeting of the photosensitizer to the desired sites. When photosensitizers are targeted to pigmented lesions in the dermis, damage to the *epidermis* can be minimized upon absorbing light of an appropriate wavelength. In PDT, the toxic, mutagenic and carcinogenic effects of photosensitizers and their photoproducts must be considered in the clinical use of the chemicals.

#### 3.1.1 Nevus of Ota

Kenji Sato and his colleagues (Sato et al., 2000) used riboflavin as a photosensitizer because it is a vitamin naturally present in normal tissue and is thought to be nontoxic at therapeutic doses. It can be targeted by injection in the skin lesions. Irradiation of riboflavin produces several reactive oxygen species, riboflavin radicals through type I reaction and singlet oxygen, superoxide, peroxide and hydroxyl radical through type II reaction (Bruice, 1984; Davis et al., 1993; Darr and Fridovich, 1994). Among them is hydrogen peroxide which has a long half-life, moves far from its production sites, and penetrates cell membranes freely (Sato et al., 1995a; Sato et al., 1995b) and thus dermal melanocytes and melanophores may be affected by hydrogen peroxide even if riboflavin does not penetrate the cell membranes. They had applied dermal injection of riboflavin and exposure to near-ultraviolet (UVA) and/or visible radiation (VR) at about solar irradiance to nevus of Ota which has many melanocytes in the dermis.

Their primary target of ribphototherapy is nevus of Ota. The purple to blue hue of the nevus is caused by a fraction of incident VR which scatters in the dermis. Riboflavin has an absorption spectrum at the blue light range and is degraded by the light. If one notices blue hue on the lesions, blue light penetrates into the point where the pigment causative of the blue hue is located. If riboflavin is there, the light produces photodynamic effects to decrease the pigment through cell necrosis or induced cell death. Therefore, theoretically, visible spots of the nevus can be treated with ribophototherapy. Thus, blue light in addition to UVA radiation is more suitable for the treatment than either of the two kinds of light alone.

### 3.2 Vascular malformations

Congenital vascular malformations are most commonly found in the head and neck and typically presenting early in life. Vascular malformations can be subdivided into lesions composing a single vessel type, such as capillary, venous and LMs, and combined lesions comprising two or more of these vessel types.

#### 3.2.1 Port-wine stains (PWS)

Port-wine stains are a type of capillary malformation (CMs) affecting 0.3%–0.5% of the population, which present mostly at birth as pink to erythematous patches on the skin and/or mucosa. However, PWS can be acquired through trauma or surgical interventions; in this case, they are referred to as acquired PWS. Histologically, PWS are characterized by ectatic capillary and venule-sized vessels within both the papillary and the reticular dermis approximately 300–600 μm below the skin surface (Viator et al., 2002). The blood vessels show inter-individually and intra-individually varying diameters ranging from 10 to 150 μm, in hypertrophic PWS up to 500 μm. Unlike vascular tumors which will mentioned later, CMs do not exhibit proliferation, but rather demonstrate chronic progressive vascular dilatation over the course of years (Laquer et al., 2013). There are many different kinds of laser treatments to deal with PWS, such as Frequency-doubled Nd: YAG laser, Pulsed dye laser (PDL), Alexandrite laser (755 nm). PWS can also be treated by intense pulsed light and photodynamic therapy.

**FIGURE 2:**
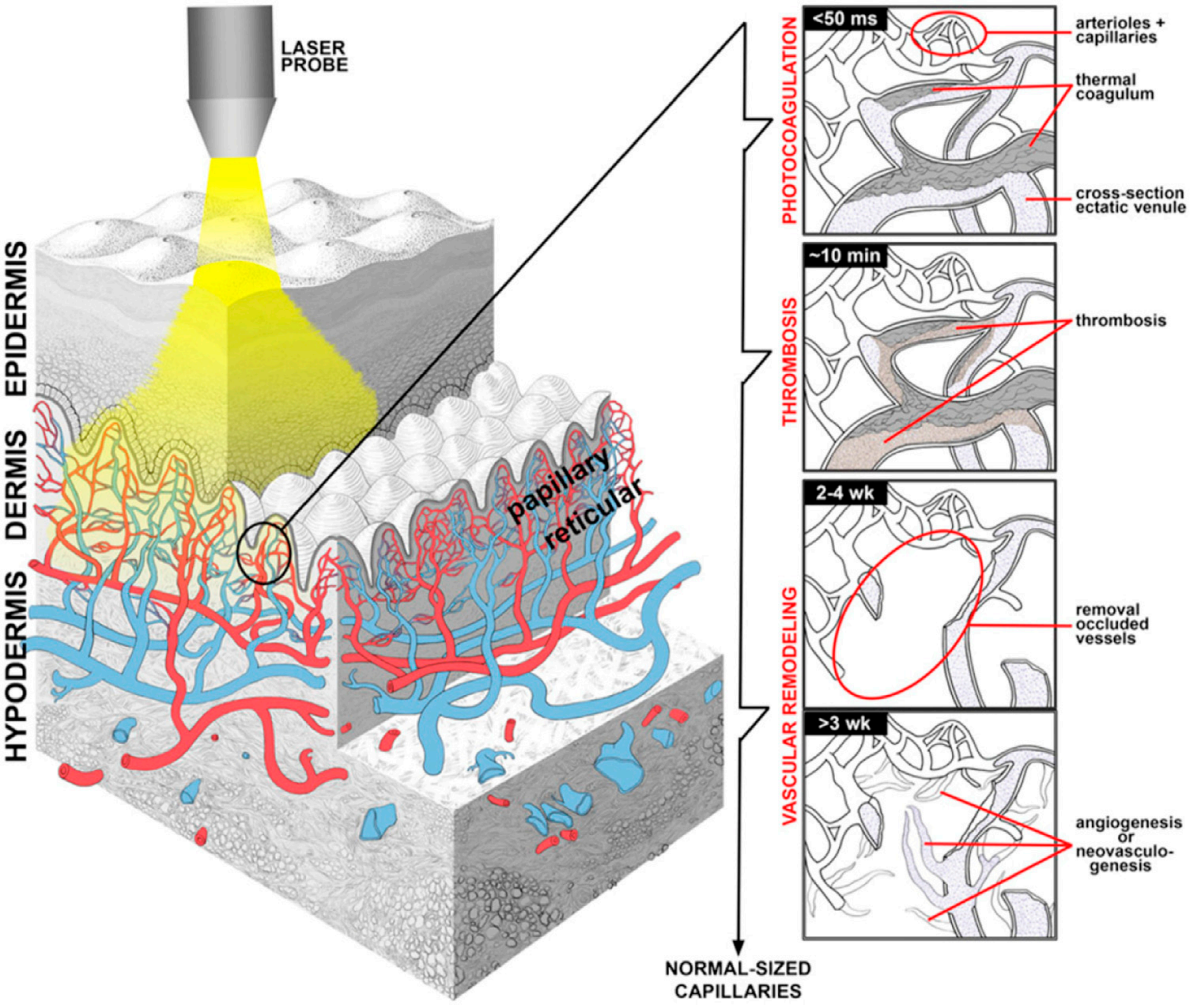
PDT treatment for vascular malformations in the field of PRS. (A) Overview of endovascular laser-tissue interactions in pulsed dye laser (PDL) treatment of refractory port wine stain (PWS) skin. Yellow light-emitting PDL is used to selectively photothermolyze ectatic venules (blue structures) in predominantly papillary dermis from Chen et al. (2012).

PDT and PDL are two most important treatments for PWS. PDT is an alternative and advancing option (Chen et al., 2012) (Figure 2). It is a relatively new therapeutic modality that shows promising results for the treatment of PWS. A chemical photosensitizer is introduced, typically via intravenous injection, and the affected area is irradiated with light of a wavelength absorbed by the photosensitizer. In the presence of oxygen, free radical damage results, with subsequent destruction of endothelial cells. One significant drawback is the side effect of generalized photosensitivity requiring photoprotection for days to weeks, depending on the half-life of the photosensitizer used. Porphyrin precursors are mostly used as chromophores with absorption peaks in the blue and red range and a smaller peak in the yellow range. Capillaries containing porphyrin derivatives can be selectively irradiated with either coherent or incoherent light of an appropriate wavelength. In combination with oxygen, the activation of the photosensitizer leads to the generation of reactive oxygen species, inducing intracapillary photothermal and photochemical effects and the subsequent destruction of the vessel wall. PDT treatment has been used alone or has been combined with other laser and light therapies in small studies. In general, studies have suggested an equivalent or possibly superior efficacy when compared to the standard PDL treatment. Indocyanine green photosensitizer activated by diode laser demonstrated efficacy in some studies (Klein et al., 2103). PDT treatment using hemoporfin and copper laser was recently studied in children 3–10 years of age and showed a higher rate of excellent response than the traditional PDL treatment. A more significant difference was seen in violaceous lesions than in those that were erythematous (Zhang et al., 2014). PDT treatment of PWS represents an emerging arena and investigators continue to explore optimization of treatment protcols (Wang et al., 2013).

Evans et al. (2005) conducted an internally controlled pilot study of eight patients with PWS comparing the efficacy of PDT using PDL as a light source to PDL (585 nm, 6.5 J/cm^2^, 7 mm) alone. The authors administered 30 mg/kg 5-aminolaevulinic acid orally followed by PDL treatment 1 or 2 h later but did not find any significant benefit in combining PDT with PDL compared to PDL alone. However, other authors found a beneficial effect in combining benzoporphyrin derivative monoacid ring PDT with PDL in contrast to PDL alone (Tournas et al., 2009). Qin et al. (2007) treated 238 Chinese patients with PWS with photocarcinorin-mediated PDT (4–5 mg/kg) using a copper vapor laser. Light was delivered at dose levels of 160–260 J/cm^2^ and fluence rates of 70–100 mW/cm^2^. The procedure was repeated 2–4 times in some patients with a follow-up of 6 months to 4 years. Excellent and good responses were obtained in 60.5% of patients, fair response in 36.6%, and poor or no response in 2.9%.

#### 3.2.2 Lymphatic malformations (LMs)

Lymphatic malformations (LMs) are benign congenital vascular malformations characterized by complexes of abnormal lymphatic vessels. There’s currently no standardized treatment approach to LMs and no established best practice in assessing disease burden or treatment effects and outcomes. Within the classification system proposed by the International Society for the Study of Vascular Anomalies in 1996, LMs are considered low-flow vascular malformations (Renton and Smith, 2011). Proposed management strategies for LMs have included surgical resection, chemical sclerotherapy, simple observation laser, photodynamic therapy, radiofrequency ablation and so on.

PDT has been studied using imaging to measure treatment effects with apparent good response and few adverse effects in at least one study (Betz et al., 2007). That study also found improvement in LM-associated symptoms such as sleep apnea and snoring and bleeding, with less improvement in pain. Another small series was similarly encouraging, reporting improved infection rates in 8 of 11 patients with LM (Jerjes et al., 2011).

#### 3.2.3 Venous malformations

As main type of low-flow malformation of the head and neck, venous malformations must be mentioned. They are the most frequently occurring malformations in humans and according to the definition they are also present at birth.

### 3.3 Inflammatory lesions

Photodynamic therapy has been used in the treatment of chronic inflammation and is an interesting alternative in the treatment of drug-resistant bacterial infections (Kharkwal et al., 2011; Sperandio et al., 2013).

#### 3.3.1 Acne vulgaris

Inflammatory acne is believed to be mainly caused by an increase in the population of Propionibacterium acnes (P. acnes). Propionibacterium acnes produce an endogenous porphyrin, coproporphyrin III, which makes it an attractive target for treatment with PDT. In addition, both Amionolevulinic acid (ALA) and MAL are strongly absorbed by the pilosebaceous unit. ALA is commonly used for PDT and when topically applied to inflammatory lesions and can be converted to protoporphyrin IX by P. acnes. protoporphyrin IX can then be stimulated by a light source to produce singlet oxygen and reactive radicals which reduce the local population of P. acnes (Hongcharu et al., 2000; Zeina et al., 2001; Williams et al., 2012). The proposed mechanisms for ALA-PDT in treating acne include the suppression of P. acnes, direct damage to the sebaceous glands and alterations of follicular keratinocyte shedding (Nestor, 2007).

Variations in the light source have been reported (use of LED, IPL, red light, blue light or combined light) as well as variations in dose and irradiation (Haedersdal et al., 2008). According to a study, which used biopsies to examine the effect of PDT on sebaceous glands, high dose (150 J/cm^2^ using 550–700 nm light source) can cause sebocyte suppression and last long (Hongcharu et al., 2000). Another study shows low dose (13 J/cm^2^ using 600–700 nm light source) can also suppress sebocyte (Itoh et al., 2001). Liu et al. (2014) compared PDT, IPL and blue-red LED phototherapy in the treatment of 150 Chinese patients with moderate to severe facial acne vulgaris and concluded that although the three methods were all efficacious in the treatment of acne, PDT required the least number of sessions. In a review of PDT studies for the treatment of acne by Sakamoto et al. (2010), the authors concluded that incubation periods of at least 3 h were associated with long-term remission, high-dose ALA-PDT and MAL-PDT (with an incubation period of at least 3 h, high fluence and red light) have similar efficacy and red light is more likely to destroy sebaceous glands than blue light or pulsed light. A 2014 evidence-based review of PDT in the treatment of acne, by Zheng et al. (2014) examined the effects and safety of PDT in the treatment of acne. They included 14 randomized controlled trials with 492 patients. The photosensitizers used included ALA, MAL, and indole-3-acetic acid (IAA), and light sources used were red light, PDL, IPL, long-pulsed dye laser (LPDL) and green light. They found that the following protocols were most efficacious in treating inflammatory acne lesions: ALA + red light, ALA + PDL, ALA + IPL, MAL + red light, and MAL + LPDL. However, ALA + red light was also effective in reducing sebum production and treating noninflammatory lesions, whereas ALA + IPL and IAA + green light was effective in significantly reducing sebum production. Moreover, triple treatment protocols were very effective in improving both inflammatory and noninflammatory lesions when compared with a 2-treatment protocol (IR LED followed by ALA + LED was compared with ALA + LED alone). Lastly, they found that the treatment efficacy could be improved by increasing ALA concentration, ALA incubation time, PDT sessions, dose of light source or occluding the photosensitizers. Professor Guoyu Zhou (Dong et al., 2016) created a new LED device with two wavelengths of light (543–548 nm and 630 ± 6 nm, respectively) for photodynamic therapy in treatment of moderate to severe acne vulgaris. The ALA-PDL treatment regimen showed an overall effectiveness rate of 89.13% and no severe adverse events were observed. Pulsed dye laser (PDL) may be used alone with ALA/MAL or in addition to red/blue light PDT. As an adjuvant, it may have particular benefit for shallow erythematous early scars from previous inflammation (Orringer et al., 2010).

#### 3.3.2 Verrucae

Several studies have demonstrated the high efficacy of PDT in the treatment of verrucae. Clearance rates of hand and foot verrucae have been reported in the range of 56%–100%. One study used red light (590–700 nm, 70 J/cm^2^) after applying ALA for 4 h showed that ALA-PDT is superior to placebo-PDT treatment of verrucae (Stender et al., 2000). Another research treated periungual and subungual verrucae with ALA-PDT. ALA was applied under occlusion for an average of 4.6 h and then irradiated with red light (580–700 nm, 70 J/cm^2^ with a range of 30–180 J/cm^2^) (Schroeter et al., 2007). They found that after an average of 4.5 treatments, total clearance was achieved in 90% of the patients. Several studies have also examined the efficacy of PDT in the treatment of genital warts. Stefanaki et al. (2003) treated males with condyloma accuminata with ALA under occlusion for 6–11 h and then irradiated with broad band visible light (400–800 nm, 70 J/cm^2^ or 100 J/cm^2^). At 1 year, the overall cure rate was 79.2%

### 3.4 Tumor

Surgery is the oldest treatment option for tumor and works best for localized tumors that have not yet spread to other parts of the body. It is often followed by radiotherapy or chemotherapy to kill the residual cancerous cells and ensure the complete removal of tumor (Diaz et al., 2003). Despite progress in basic research that has given us a better understanding of tumor biology and led to design of new generations of targeted drugs, recent large clinical trials for tumor, with some notable exceptions have been able to detect only small differences in treatment outcomes (Bergh, 2009). Moreover, the number of new clinically approved drugs is disappointingly low (Hampton, 2006) and these treatments are expensive. The main challenges in the tumor treatments are severe side effects when treatment affects healthy tissues or organs. These sobering facts indicate that to make further progress it is necessary to put an emphasis on other existing but still underappreciated therapeutic approaches. There is an urgent need for developing accessible and affordable tumor treatment modalities.

**FIGURE 3:**
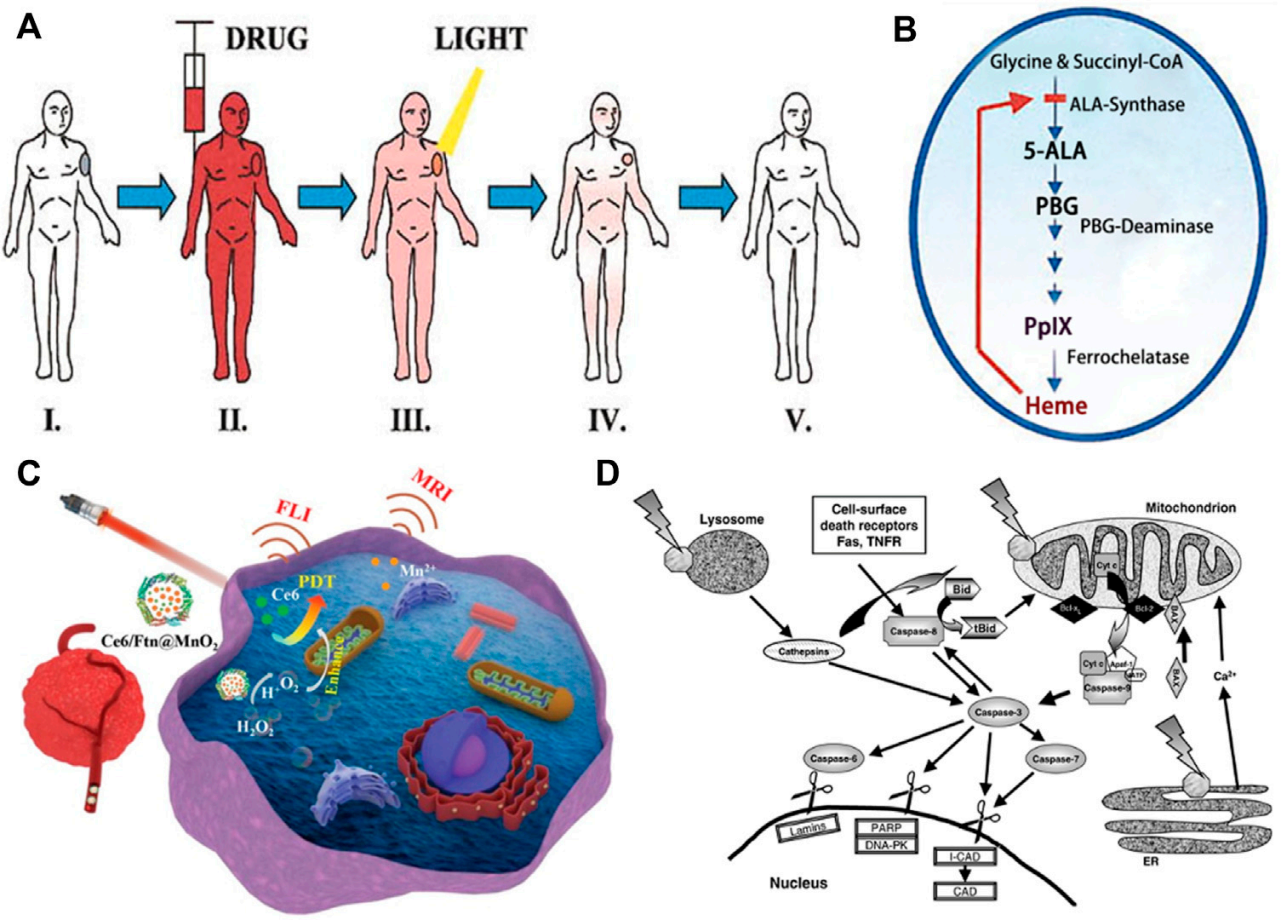
PDT treatment for tumor in the field of PRS. **(A)** PDT treatment procedure. Step I: Patient with tumor before PDT. Steps II-III: The photosensitizer is taken up by most tissues, but it is retained longer in the tumor. The malignant lesion is irradiated selectively at the maximal tumor-to-normal-tissue drug ratio. Step IV: The photosensitizer clears gradually from all tissues while the tumor starts to shrink as a result of PDT-mediated tumor cell destruction. Step V: Complete tumor ablation and clearance of photosensitizer from Kalka et al. (2000). **(B)** Porphyrin metabolism under physiologic conditions in tumor cells during ALA-based PDT from Kalka et al. (2000). **(C)** Schematic illustration of Ce6/Ftn@MnO_2_ used for FLI and MRI bimodal imaging-guided hypoxic malignant tumor therapy from Zhu et al. (2022) **(D)** Some PDT-associated apoptosis pathways involving plasma membrane death receptors, mitochondria, lysosomes and ER, caspases and Bcl-2 family proteins. Most photosensitizers for PDT bind to mitochondria, lysosomes and/or other intracellular membranes, including the ER from Oleinick et al. (2002).

Though PDT was discovered nearly 100 years ago, total number of approved drugs is still limited and the technique remained unappreciated and underutilized. The emerging extensive research in this field would provide the impetus in making PDT as a potential therapeutic modality for managing malignant and non-malignant tumors. Although still emerging, it is already a successful and clinical approved therapeutic modality used for the management of neoplastic and non-malignant diseases. The use of PDT for treatment of various human skin cancers was first investigated in the 1970s by Dougherty (Dougherty et al., 1978a). It is a minimally invasive therapeutic technique used in the management of various cancerous and pre-malignant diseases. The clinical use of PDT for tumor dates to the late 1970s when there was a study on the effects of HPD + light in five patients with bladder cancer (Kelly and Snell, 1976). In 1978, Dougherty reported the first large series of patients successfully treated with PDT with HPD (Dougherty et al., 1978b). The procedure involves administration of a photosensitizing agent followed by irradiation at a wavelength corresponding to an absorbance band of the sensitizer. In the presence of oxygen, a series of events lead to direct tumor cell death, damage to the microvasculature and induction of a local inflammatory reaction. Clinical studies revealed that PDT can be curative particularly in early-stage tumors. It can prolong survival in inoperable cancers and significantly improve quality of life. For malignant tumor, hypoxia is one of the hallmarks, which induces an unexpected resistance of tumors to PDT since molecular O_2_ plays an essential role during the process. Pro. Liu constructed an O_2_ self-supply PDT system by co-encapsulation of chlorin e6 (Ce6) and a MnO_2_ core in an engineered ferritin (Ftn), generating a nanozyme promoted PDT nanoformula (Ce6/Ftn@MnO_2_) for tumor therapy (Zhu et al., 2022). This method enhanced the effectiveness and precision of PDT by TME modulation (Figure 3C).

PDT using Photofrin and ALA and its derivatives has been extensively studied in the treatment of both premalignant and malignant skin tumors (Braathen et al., 2005). We will discuss PDT of tumor treatment in the field of PRS (Figures 3A,B,D). There are many typical kinds of tumors, such as melanoma, neurofibroma, basal cell carcinoma, squamous cell carcinoma and vascular tumors.

#### 3.4.1 Melanoma (MM)

Melanoma is a tumor produced by melanocytes of skin and other organs. It originates from the melanocytes in the basal layer of *epidermis* (Williams et al., 2011). Although MM represents a small percentage of skin cancers, it causes the majority of skin cancer related deaths due to its high potential for invasion and metastasis (Ward et al., 2019). The incidence of melanoma has increased sharply in the past few years, with high degree of malignancy, poor prognosis, easy local recurrency and metastasis. It is growing at a rate of 3%–5% every year in our country (Guo et al., 2015). Common skin melanoma can be divided into superficial diffuse type, nodular type, acral freckle type and malignant freckle type. Acral freckle type is most common in China (Guo et al., 2017). In the past decades, the main clinical treatment options were chemotherapy, radiotherapy and surgery (Ferrari, 2005). Due to the feature of aggressive and metastatic of melanoma, it is challenging for traditional therapies to make removal of entire tumor tissue and avoiding a high risk of recurrence. Drug resistance and low immunotherapeutic activity remain major challenges for emerging target and immunotherapy therapies. Therefore, it is very important to develop effective alternative treatment strategies for melanoma.

Photodynamic therapy is a clinically approved, minimally invasive therapeutic procedure that can exert a selective cytotoxic activity toward malignant cells.

#### 3.4.2 Hemangiomas

Hemangiomas is one of typical kind of vascular tumors. It affects one in ten children and most are uncomplicated, resolving spontaneously in 3–5 years. According to some reports, most hemangiomas (70%–90%) occurred in the first month after birth (Acevedo and Cheresh, 2008). Occurrence rate in the 12th month decreased to 10%–12%. Hemangiomas can be divided into nine types based on clinical manifestations, including erythema, strawberry birthmarks, tumor, ulcer, scar, dermatitis eczema, Carmel syndrome, mixed and systematic type. Among them, the erythema type is most common in infant clinic. These benign vascular tumors are usually uncomplicated and tend to regress spontaneously. However, when hemangiomas occur in high-risk areas, such as near the eyes, throat or nose, impairing their function or when complications develop, intervention may be necessary (Boscolo and Bischoff, 2009). Most hemangioma can pass rapid growth period, relatively stable period and finally enter regression period. Because of the diversity and complexity of different types of hemangiomas, most of them cannot be completely resolved and still require laser treatment or surgical intervention (Zaher et al., 2016; Prabhuraj et al., 2019).

Photodynamic therapy has been successful in treating hemangiomas.

#### 3.4.3 Basal cell carcinoma (BCC)

Basal cell carcinoma is the most common non-melanoma skin cancer and its incidence continues to rise. Traditional treatments for BCC typically consist of simple excision, electrodesiccation for low-risk lesions, Mohs’ micrographic surgery (MMS), topical chemotherapeutic agents and immunomodulators (Silverman et al., 1992; Griffiths et al., 2005). Photodynamic therapy also has emerged and shows promising results.

The mechanism of PDT has fully described before. In addition to direct cytotoxicity, vascular damage resulting in tissue infraction, apoptosis through stimulation of multiple signal transduction reactions and upregulation of immune mediators, such as IL-1 and IL-6, have been shown to further promote tumor destruction (Engbrecht et al., 1999; Oleinick et al., 2002). Multiple studies have investigated the short-term efficacy of topical PDT for the treatment of BCC, although well designed studies with randomisation, double-blind analysis, post-treatment histopathology, and long-term follow-up are limited. Moreover, many studies lack specific endpoints and instead utilise clinical observations to assess efficacy. While response rates for superficial BCC range from 79% to 100% with good cosmesis, lower rates of <50% have been reported for pigmented and morphoeic BCCs, and recurrence rates of up to 63% have been reported (Kennedy et al., 1990; Kalka et al., 2000). However, another study has suggested longer-term maintenance of cure rates at 1 year post-treatment (Soler et al., 1999). Postulated reasons for lower efficacy rates for other subtypes when compared with superficial BCCs include inability to administer light to deeper tumor depths and absorption of photoactivating light by melanin (Lui et al., 1995; Itoh et al., 2000). It has also been suggested that tumor thickness and subsequent depth of photosensitizer penetration may be an important determinant of efficacy (Morton et al., 1998). Adverse events after topical photodynamic therapy most commonly consist of local site reactions, including burning, stinging, crusting, erythema, and transient pigmentary changes at the treatment site. Local reactions, transient and mild in severity, were reported in as many as 79% of patients that underwent photodynamic therapy (Vinciullo et al., 2005). Higher pain scores have been reported for 5-aminolaevulinic acid compared with 5-aminolaevulinic methylester (Wiegell et al., 2003). Systemic administration is associated with prolonged photosensitivity and adverse effects such as hyperthermia, nausea, vomiting, headache and myalgias (Brown et al., 2004). Hence, following administration of photosensitiser, exposure to sunlight or bright in-door light should be avoided. Wang et al. (2001) reported a shorter healing time with aminolaevulinic acid- PDT than with cryotherapy, as manifested by less leakage and oedema. Rarely, allergic reactions to the porphyrin component may occur, and caution must be exercised in patients with known photosensitivity disorders, porphyria or porphyrin allergies.

Topical PDT has shown promising results with good cosmesis in the treatment of BCC in a prospective, randomised, double-blind, vehicle-controlled study, although most studies investigating PDT were nonrandomised and relied on clinical observations for efficacy analysis (Foley, 2003). However, high recurrence rates (up to 63%) have been reported with longer-term follow-up. PDT may be useful in the treatment of superficial BCCs, particularly in those patients who are not amenable to surgery because of large lesion size or multiple lesions, although treatment of larger skin areas may result in increased pain (Grapengiesser et al., 2002). Adverse events are typically local site reactions and generally mild in severity. Additional studies with randomised, double-blind design and long-term follow-up are needed to better characterise the efficacy and safety profile of photodynamic therapy for BCC, particularly with respect to aggressive subtypes, adjunctive methods seeking to enhance tissue penetration, optimal number of treatments, and best time interval between drug and exposure to light. Standardised investigation of time intervals may prove important, given that studies have shown that a short interval promotes accumulation of sensitiser primarily in the intravascular compartment, resulting in thrombus formation and indirect killing (Dolmans et al., 2002a; Dolmans et al., 2002b). A longer duration, on the other hand, may promote localisation to the extravascular compartment via vascular leakage and interstitial diffusion. Until these considerations are addressed in further studies, PDT should be considered investigational and limited to the treatment of superficial BCCs in low-risk areas where recurrence is unlikely to result in significant morbidity.

#### 3.4.4 Squamous Cell Carcinoma (SCC)

PDT has demonstrated efficacy in treating squamous cell carcinoma *in situ*/Bowen’s disease and has also been used with some success to treat extramammary Paget’s disease. However, the results of PDT for squamous cell carcinoma of the skin using topical photosensitizers have been disappointing, with recurrence rates of >50% (Nestor et al., 2006).

Current studies focus on novel photosensitizer drugs and re-formulations of ALA, such as nanoemulsion or patch based applicators, that may increase the complete response rate for AK at 12 months to >95% (Szeimies et al., 2010). Bowen disease is defined as full-thickness epidermal atypia without invasion into the dermal layer. The results of ALA-PDT in the treatment of Bowen’s disease (squamous cell carcinoma *in situ*) have been equally positive and so far were reported in 6 randomized clinical trials. Randomized, controlled trials comparing ALA-PDT or MAL-PDT to cryotherapy or 5-FU cream reveal complete response rates of 82%–100% for PDT versus 67–100% for cryotherapy or 79–94% for 5-FU at 12–24 months (Morton et al., 1996; Morton, 2007). Morton et al. (2006) conducted several clinical trials to optimize ALA-PDT for the treatment of Bowen disease. The authors concluded that red light (630 nm) was superior to green light (540 nm) in both complete clearance and recurrence rates. Furthermore,Salim et al. (2003) compared ALA-PDT with topical 5-FU in the treatment of Bowen disease. ALA was incuvated for 4 h followed by irradiation with narrow-band red light (630 nm, 100 J/cm^2^). Both ALA-PDT and 5-FU were repeated 6 weeks later as necessary. One year later, they found that 82% of the lesions treated with PDT showed complete response versus 42% with 5-FU.

### 3.5 Obesity

Obesity is an increasingly serious public health issue and also a really important problem in the field of plastic surgery, which can affect both health and appearance. Photodynamic therapy and adipose browning induction are two promising approaches to reverse obesity. Professor Liming Nie from Xiamen University has reported on nature nanotechnology that combining a traceable photosensitizer zinc phthalocyanine tetrasulfonate (ZnPcS4), and a browning agent (rosiglitazone) to hepatitis B adipose-targeted protein complex structure. This structure can perform photodynamic treatment, fat browning and photoacoustic molecular imaging simultaneously.

### 3.6 Cosmetic

#### 3.6.1 Actinic keratosis

In the United States, the only FDA-approved indication for PDT in 1999 is the treatment of nonhyperkeratotic AKs on the face and scalp in conjunction with a blue light source (Babilas et al., 2010). The initial FDA Phase II and III studies of ALA-PDT (Levulan Kerastick, DUSA) in the treatment of nonhyperkeratotic actinic keratosis on the face and scalp had a clearance rate of 85%–90% after one to two treatment sessions. The ALA was applied for 14–18 h, followed by irradiation with a blue light source (10 J/cm^2^) for 1,000 s. A recent Cochrane review found that ALA-PDT or MAL-PDT with blue or red light, resulted in similar efficacy in the treatment of actinic keratosis. However, for ALA-PDT, longer incubation (4 h) resulted in better results compared with shorter incubation time (<2 h) (Gupta et al., 2012). The review also found that 4 h incubation with ALA-PDT was significantly more efficacious than cryotherapy, but 1 h incubation with ALA-PDT (blue light or pulsed dye laser) was not significantly different than 0.5% 5-FU. Tierney (Tierney et al., 2008) assessed patient perceptions and preferences in the management of actinic keratosis and found that PDT had faster recovery and improved cosmetic outcome when compared with surgical excision and cryotherapy. Furthermore, patients preferred PDT to 5-FU or imiquimod.

#### 3.6.2 Actinic cheilitis

Although actinic cheilitis is a cosmetic problem, it is also a premalignant condition localized to the lips, which can progress to invasive squamous cell carcinoma (SCC), similar to AKs on the skin. Treatment of actinic cheilitis of the lip with PDT has expected outcomes which is equal to or exceed other FDA-approved treatment modalities. Fractional ablative laser pretreatment improves outcomes in all published studies. Many patients who have previously undergone treatment with cryotherapy, topical 5-FU or imiquimod ultimately prefer PDT. This may be due to decreased peak pain compared with cryotherapy, shorter total treatment and recovery time compared with many topical agents and improved cosmetic outcomes.

In 2015, Yazdani abyaneh et al. (2015) completed a systemic review that examined the treatment of actinic cheilitis with PDT. Both ALA and MAL were used with incubation periods that ranged from 2 to 4 h. Red light (630 nm) irradiation was used for both photosensitizers, ranging 37–80 J/cm^2^. The studies that examined histological clearance found a cure rate of 47% with a follow-up period ranging from 1.5 to 18 months. The most common reported adverse events were pain and burning, which resolved within 2 weeks. Alexiades-Armenakas and Geronemus (Alexiades-armenakas and Geronemus, 2004) reported in 2004 using laser-mediated PDT for actinic cheilitis. They treated 19 patients with a 595-nm pulsed dye laser (7.5 J/cm^2^, 10 mm spot, and 10 ms pulse duration). One to 3 treatments were given in 1-month intervals. The complete response rate was 13/19 or 68% after a mean of 1.8 treatments.

#### 3.6.3 Photorejuvenation

Multiple clinical studies have consistently demonstrated good to excellent cosmetic results with the use of PDT.

Babilas et al. (2008), in a prospective, randomized controlled, split-face study, treated 25 patients with sun-damaged skin, treated with MAL, followed by irradiation with either an LED (635 nm, 37 J/cm^2^) or an IPL device (610–950 nm, 80 J/cm^2^). At 3 months, the authors found significant improvement in wrinkling and pigmentation, irrespective of the light source used. Gold et al. (2006) evaluated short-contact (30–60 min) ALA-PDT using IPL as a light source, versus IPL alone in 16 patients in a side-by-side design. Patients were exposed to PDT for a total of 3 monthly treatments and followed at 1 and 3 months. The IPL treatment parameters were 34 J/cm^2^; cutoff filters used were 550 nm for Fitzpatrick skin Types I to III and 570 nm for Fitzpatrick skin Type IV. They found greater improvement in the ALA-PDT-IPL group, compared with IPL alone for all facets of photodamage.

The practical challenge with using PDT for photorejuvenation is the availability of multiple other proven and accepted modalities such as chemical peels, laser and IPL. The additional time and supply costs of PDT limit it from becoming a widely used treatment option for photorejuvenation.

Following list most common applications in the field of Plastic and Reconstructive Surgery mentioned before (Table 2).

**TABLE 2:** Photodynamic therapy specific treatment protocols for most common applications in the field of plastic and reconstructive surgery.

Application	Topical photosensitizer	Incubation period	Light source	Dose
PWS	ALA	1–2 h	Red light	160 J/cm^2^-260 J/cm^2^
Acne Vulgaris	ALA	3 h	Blue light	10 J/cm^2^
	MAL	3 h	Red light	37 J/cm^2^
			Red light	37 J/cm^2^
Verrucae	ALA	4–5 h	Red light	30–180 J/cm^2^
BCC	ALA	3–6 h	Red light	≥60 J/cm^2^
	MAL	3 h	Red light	35 J/cm^2^-75 J/cm^2^
SCC	ALA	4 h	Red light	≥100 J/cm^2^
	MAL	3 h	Red light	75 J/cm^2^-100 J/cm^2^
Actinic Keratosis	ALA	1–4 h	Red light	75 J/cm^2^-150 J/cm^2^
			Blue light	10 J/cm^2^
	MAL	0.5 h	Daylight	2 h
		1–3 h	Red light	37 J/cm^2^-75 J/cm^2^
Actinic Cheilitis	ALA	2–4 h	Red light	37 J/cm^2^-80 J/cm^2^
Photorejuvenation	MAL	30 min-3 h	Blue light	10 J/cm^2^
	ALA			
			Red light	37 J/cm^2^
	MAL	30 min-1 h	Red light	37 J/cm^2^

ALA, aminolevulinic acid; BCC, basal cell carcinoma; MAL, methyl aminolevulinate; SCC, squamous cell carcinoma; PWS, Port wine stain.

## 4 Limitation

The use of PDT may still have many practical problems, such as prolonged systemic visible light sensitivity after intravenous administration of porphyrin derivatives. As we know, the most commonly reported adverse events associated with the PDT treatments were pain, burning or an itching sensation of the skin, erythema and edema, which generally resolved within several hours and were tolerated. What’s more, post-inflammatory hyperpigmentation was normal after PDT treatment and it usually resolved within several months.

## 5 Conclusion and outlooking

Photodynamic therapy is based on the possibility of the selective destruction of pathological tissues with accumulated photosensitizer. Comparing to other therapeutic methods in oncology, PDT is distinguished by its selectivity with equivalent therapeutic results. PDT can also combine with other kinds of treatments. For example, the combination of PTT and PDT with other therapeutic modality, leading to additive or even synergistic therapeutic effects. Nanotechnology might play a prominent role in combination therapy approaches because nanoplatforms present a vehicle for the integration of various agents associated with different therapeutic paradigms. However, several proposed combination partners for photodynamic therapy, such as chemotherapy or immunotherapy are already well-established, standard-of-care treatments. Therefore, initial clinical studies are likely to involve the combination of photodynamic therapy with currently approved treatments. Cooperative interactions between different therapies could potentially increase the treating efficacy at lower doses of photosensitive agents or lower-power light irradiation, thus minimizing potential toxicity to healthy tissues.

Photodynamic therapy is gaining more and more prominent position not only among clinicians, but especially among patients. It is considered to be a new and promising strategy in the field of PRS. Its full potential has yet to be shown and its range of applications alone or in combination with other approved or experimental therapeutic approaches is definitely not exhausted. The advantages of PDT compared with surgery chemotherapy or radiotherapy are reduced long-term morbidity and the fact that PDT does not compromise future treatment options for residual or recurrent disease. Rapid development and advances in technology and optoelectronic equipment can favor the implementation of the photodynamic method in clinical practice in the field of plastic and reconstructive surgery.
